# Physiotherapy in Indian communities: a brief review

**DOI:** 10.15171/hpp.2017.21

**Published:** 2017-06-14

**Authors:** Pavithra Rajan

**Affiliations:** Shastri Indo-Canadian Institute, Canada, Fellow, Shastri Indo-Canadian Institute, Calgary, Canada

**Keywords:** Home care services, Exercise, Home visits, Holistic health

## Abstract

**Background:** Importance of community rehabilitation in India has been emphasized in previous research. There is ample research that has been published for different communities in the country. However, the precise role of physiotherapy in community rehabilitation is unclear.The objective of the current brief report is to look into the role of physiotherapy in community rehabilitation.

**Methods:** Relevant literature search was done using databases namely Medline, Scopus, PubMed, PEDro and CINAHL using search terms- India, community rehabilitation, home rehabilitation, home exercises and physiotherapy. Studies that followed the PICO format, published in English,after 2005 and that had specifically mentioned the role of physiotherapy in community projects were included.

**Results:** While there are handful of studies that have mentioned the contribution of physiotherapy in the community, most of the interventions are targeted toward management of chronic health conditions. More work needs to be done to outline the importance and precise role of physiotherapy in the rehabilitation of communities in India, especially in preventive care.A model has been created to emphasize the holistic approach of physiotherapy in the Indian setting.

**Conclusion: ** Physiotherapy has a pivotal position in community rehabilitation in India.However, published research for the same is lacking. While physiotherapy interventions have been designed to target chronic health conditions in the community, emphasis on preventive care is lacking.

## Introduction

India is an interesting country. As per the World Health Organization (WHO), with a population of 1.295 billion and still growing, public health expenditure sums to 30%, which includes expenditure on curative as well as preventive services, among others. Community based rehabilitation (CBR) has been viewed as a good alternative to the costly institution based rehabilitation since many decades in India, though it can be challenging especially in resource constraint settings.^1,2^

There have been country-wide community rehabilitation by skilled professionals for selected health conditions in India like trained caregivers for stroke,^3,4^ trained workers from community for psychosis,^5^ speech language pathology services for children with cleft palate,^6^ ophthalmologist trained community workers for the blind,^7^ trained workers for persons with disabilities,^8-10^ medical staff for neuritis,^2^ home care advisors who were supervised by a counselor and a psychiatrist for dementia,^11^ leisure specialists for spinal cord injured community dwellers,^12^ mental health workers trained from the community for schizophrenia,^13^ community health workers for acute coronary syndrome,^14^ with the need for CBR for other health conditions.^15,16^ Nevertheless, “because CBR cannot be described as a discrete intervention, and the expected outcomes are not standardised, its effectiveness is hard to establish.”^1^ In addition, it has been noted that research in CBR is seldom published by the low and middle income countries,^1,17,18^ where the need for this type of rehabilitation can be anticipated to be more; however there is ample work on institution based rehabilitation. There have been conflicting evidence comparing the institution versus CBR in India.^19^ CBR for each community is unique, since local factors like culture, coping mechanisms of the community, level of awareness, motivation, and acceptability of a suggested method of treatment could affect the outcome.^20^ Over the years, CBR models are usually those that have been developed or replicated from a foreign model and implemented by local bodies like NGOs and hospitals.^21^ There are ample studies looking at different prevention strategies at the community level to reduce healthcare costs; physiotherapy in particular has been shown to be cost effective in various health conditions, as per the review by Peterson et al.^22^ Although importance of physiotherapy in CBR is known,^23,24^ inclusion of physiotherapy in CBR is hardly documented.

The National Programme for Rehabilitation of Persons with Disabilities (India) was piloted in the year 2001-2002.^25^ Although this program was targeted for the disabled communities, the benefits of integration of institution and CBR for different health conditions cannot be emphasized more. This integration of health services to treat patients holistically could be used especially by physiotherapists in India, since there is an increasing need for multidisciplinary approach to community issues. The documented evidence of role/involvement of physiotherapy in CBR, especially in low and middle income countries, is seldom,^26^ although physiotherapy could help communities in more than one ways.^27,28^ The first person account by Ward talks about how preventive medicine was not given much priority in the villages in India, and how preventive medicine could prove cheaper than curative health services.^29^ Although this paper dates back to more than 40 years, certain lacunae in community based physiotherapy still hold true to date. There is limited documentation on the impact of physiotherapy in community health programmes in India. Hence, it was of interest to understand the effectiveness of physiotherapy in community care.

The aim of the current paper is to study the effectiveness of physiotherapy services for the community in India; secondarily a holistic model has been suggested for use in integrated rehabilitation of the patients and their communities.

## Methods

Relevant literature search was done using databases namely Medline, Scopus, PubMed, PEDro and CINAHL using search terms- India, Community rehabilitation, home rehabilitation, home exercises and physiotherapy. Studies that followed the PICO format published in English and after 2005 were included. Only those studies that looked exclusively at physical therapy services in the community were included. It was of interest to understand the involvement of physiotherapy in community rehabilitation over at least a decade and hence studies published after 2005 were included in this review. The search was conducted in June 2016 (see Table 1).

## Results

To date since 2005, less than 10 studies have exclusively looked into the effectiveness of physiotherapy services in community rehabilitation in India (see Table 1). Physiotherapy has proved effective for chronic health conditions like cancer, stroke, fibromyalgia and multiple disabilities. However, the preventive aspects of physiotherapy for acute community rehabilitation have not been studied.

## Discussion

**Figure 1:**
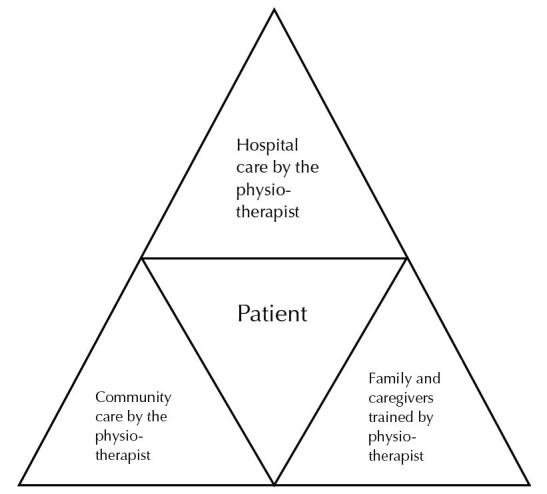
Model of holistic physiotherapy care for communities in India.

Community physiotherapy is an emerging field and India has come a long way with rapid developments in this field over the past few decades.^35^ It is evident that communities could benefit from physiotherapy services. However, emphasis on the community approaches to health care is in use only for a handful of diseases like cancer, stroke and fibromyalgia. Musculoskeletal disorders are on the rise in India, including diabetes, cardiovascular conditions among others. There is a need for a holistic approach to physiotherapy services for the community in India, especially for prevention. Preventive community physiotherapy can help in reducing the healthcare burden. As pointed out by Arora,^25^ an integrated health care system needs to be developed for communities. The role of a physiotherapist at various levels of community rehabilitation needs attention (Figure 1).

A model has been proposed based on the author’s personal experiences and many years of study of physiotherapy services in India. This model (Figure 1) shows integration of physiotherapy services at both institution and community levels, possibly assuring a holistic approach to treatment of communities. Hospital is most often the primary contact for a patient and through the patient, the physiotherapist could get access to his/her community. Hence the initial contact at the institution level is important to initiate preventive as well as curative services for the community. Based on personal experiences of the author and interactions with physiotherapists in the country, it is likely that this model could be already in use for rehabilitation in India. However, the documentation and evidence of the same is lacking. Future studies need to look into the feasibility and effectiveness of this model.

## Limitation

The current review was brief. It was limited to studies in India.

## Recommendation

Future research could include a systematic review including other developing countries.

## Conclusion

Physiotherapy has a pivotal position in community rehabilitation in India. However, published research for the same is lacking. There is seldom published research in this regard. A holistic approach to health care of communities needs to be incorporated into the existing models of physiotherapy services in India, with focus not only on curative services for chronic health conditions but also for preventive care.

## Ethics approval

Not applicable

## Competing interests

The author declares no conflict of interest. The author claims no part of this paper copied from other sources.

## Funding

Nil.

**Table 1:** Physiotherapy services in home and community in India

**Title**	**Health condition**	**Objective**	**Type of study**	**Sample size**	**Physiotherapy Intervention**	**Outcome**	**Limitations**
Effect of home-based exercise program on lymphedema and quality of life in female post mastectomy patients: Pre-post intervention study^30^	Lymphedema post mastectomy	To evaluate the effects of a home-based exercise program on lymphedema and QOL of breast cancer patients	Pre-post intervention study	n = 32	Patient education Home exercises supervised initially by the physiotherapist, later exercise handout and logbook; weekly telephonic monitoring	Improved Quality-of-Life and reduced swelling in the upper limbs	Small sample size, no control group
*Effectiveness and cost-effectiveness of telephone-based support versus usual care for treatment of pressure ulcers in people with spinal cord injury in low-income and middle-income countries: study protocol for a 12-week randomised controlled trial^31^	Pressure ulcers post spinal cord injury	To determine the effectiveness and cost-effectiveness of telephone-based support to help people with spinal cord injury manage pressure ulcers at home in India and Bangladesh	Multicentre, prospective, assessor-blinded, randomised controlled trial	n=120	Control group: usual community care and an information pamphlet Intervention group: In addition to above care, they will receive 15-25 min/ week for 12 weeks telephonic consultation with a trained physiotherapist or nurse on pressure ulcer management	Primary: Size of pressure ulcer Secondary: Severity, depth and undermining distance of pressure ulcer, depression, quality of life	Clinicians and patients not blinded
Effect of amitriptyline vs. physiotherapy in management of fibromyalgia syndrome: What predicts a clinical benefit?^32^	Fibromyalgia	To compare physiotherapy and amitriptyline for disability reduction in patients with fibromyalgia & to determine which clinical features at baseline would predict benefit with either therapy	Different treatment allocation to both groups	n=156 (82: amitriptyline; 74: physiotherapy)	Structured physical training-relaxation, stretching and strengthening exercises, Aerobic exercises, At least 10 minutes of home exercises daily, Monthly follow ups for 6 months	Decreased scores on Fibromyalgia Impact questionnaire in both groups after 6 months	Small sample size No control group
FAmily-Led RehabiliTaTion aftEr Stroke in INDia: the ATTEND pilot study^4^	Stroke	To check the feasibility of conducting a large-scale study of family-led, trained caregiver-delivered, home-based stroke rehabilitation	Randomized controlled trial	n = 104	Control group: usual community care Intervention group: a stroke rehabilitation package of care that starts in hospital and continues at home by the caregiver who is trained by an experienced nurse or physiotherapist, including regular home visits for 2 months- telephone follow ups if home visits are not possible	It is feasible to conduct a trial using an intervention of an empowered caregiver to give home-based rehabilitation after stroke	
*Family-led rehabilitation after stroke in India: the ATTEND trial, study protocol for a randomized controlled trial^3^	Stroke	To test the effectiveness of a family-led caregiver-delivered home-based low-cost rehabilitation intervention for stroke patients in India	Randomized controlled trial	n = 2400	Same as above	Home based rehabilitation after hospital discharge will be safe and cost effective	
Impact of physical therapy on burden of caregivers of individuals with functional disability^33^	Chronic disabling health conditions	To evaluate the effects of a tailored physical therapy intervention, or caregiver education, on the caregivers’ burden	Randomized controlled trial	n=66 (Control: 24, intervention: 21; caregiver education: 21)	Control group: no treatment Intervention group: 1 month tailored intervention-3-5 days a week for 20-60 minutes Caregiver education: only caregiver education; 2 days a week-10-30 minutes	Significant reduction in all three groups; no significant findings between groups however trends of reduced caregiver stress and burden in intervention group	Heterogenous groups, Telephone interviews for follow up may have biased results
Effect of CDT and home program on health- related quality of life in post mastectomy lymphedema patients^34^	Lymphedema post mastectomy	To evaluate the effect of adding an exercise component and a home program to complete decongestive therapy (CDT) on Health related quality of life in post mastectomy lymphedema patients	Randomized controlled trial	n = 60 (30 in each group)	Control group: manual lymphatic drainage, low elastic compression garment, glenohumeral mobilization, deep breathing exercises and massage strokes; 5 times a week for 6 weeks. Intervention group: manual lymphatic drainage, compression garment worn 23 h daily, remedial exercises and a home program(self -lymphatic drainage, skin care and the remedial exercises) by a trained physiotherapist, including information booklets and logs.	There was improvement in pain (as measured using Visual Analogue Scale) and quality of life (as measured using European Organization of Research and Treatment for Cancer) in both groups, intervention more than control.	Small sample size, No long-term follow up, Lack of random assignment

*In progress.
